# Supplementary figures and images for: Development and validation of HIV SMRTcap for the characterization of HIV-1 reservoirs across tissues and subtypes
Source: PLoS Pathog. 2026 Jan 13;22(1):e1013171. doi: 10.1371/journal.ppat.1013171 (PMC12851485; doi:10.1371/journal.ppat.1013171)

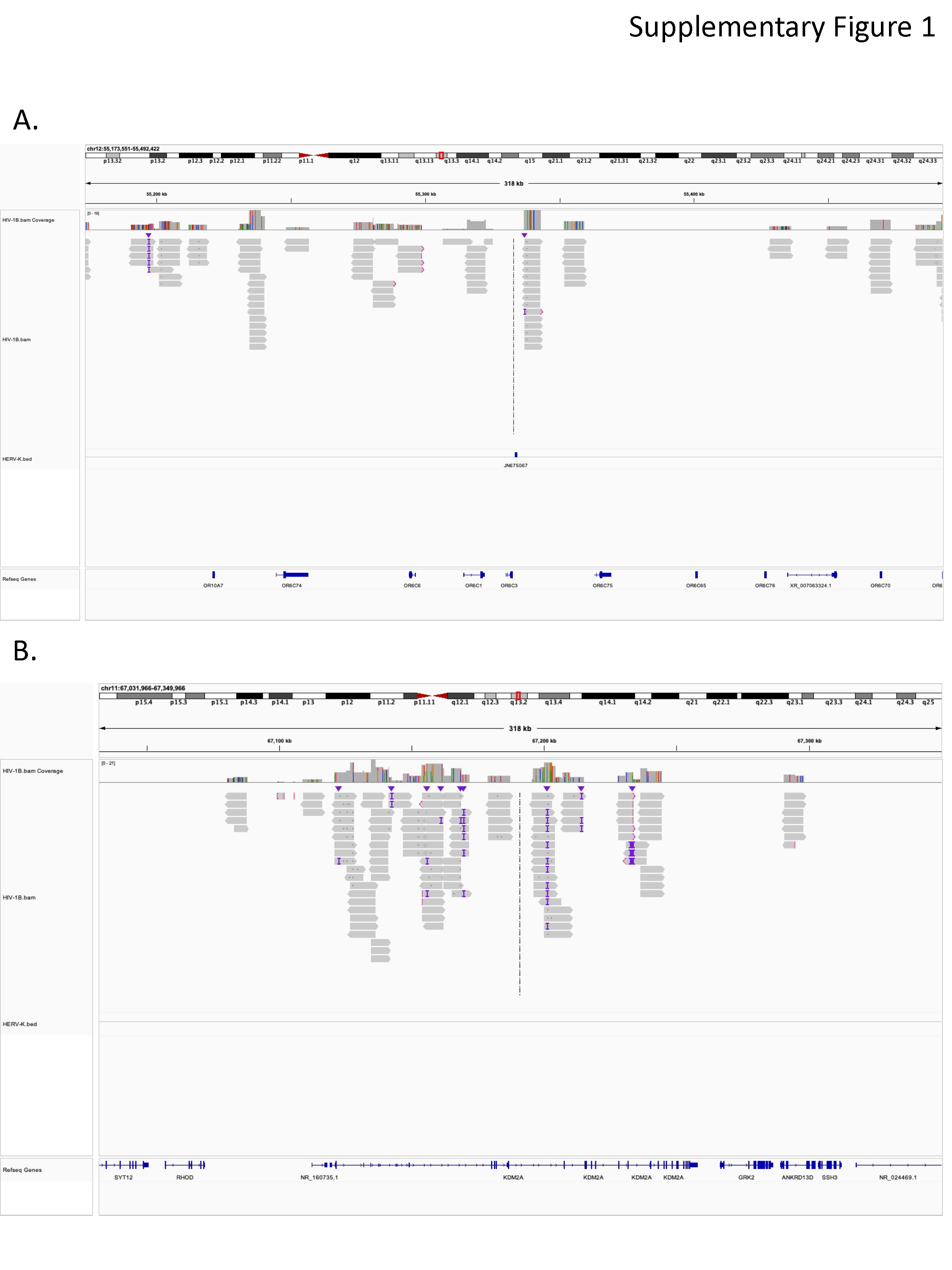

Supplement: S1 Fig — (A) Alignment of off-target (e.g., non-HIV-containing) reads to a gene-rich region of the human genome, demonstrating an average coverage between 0–16-fold. (B) Alignment of off-target reads to human endogenous retrovirus K, known to be integrated at chr 11 in q13.2 (exact position identified by vertical black dashed line) and surrounding non-viral content. Average coverage of entire fragment is between 0 and 21-fold, similar to that observed in non-lentiviral containing regions of the human genomes. (TIFF) [file ppat.1013171.s001.tiff]

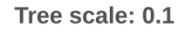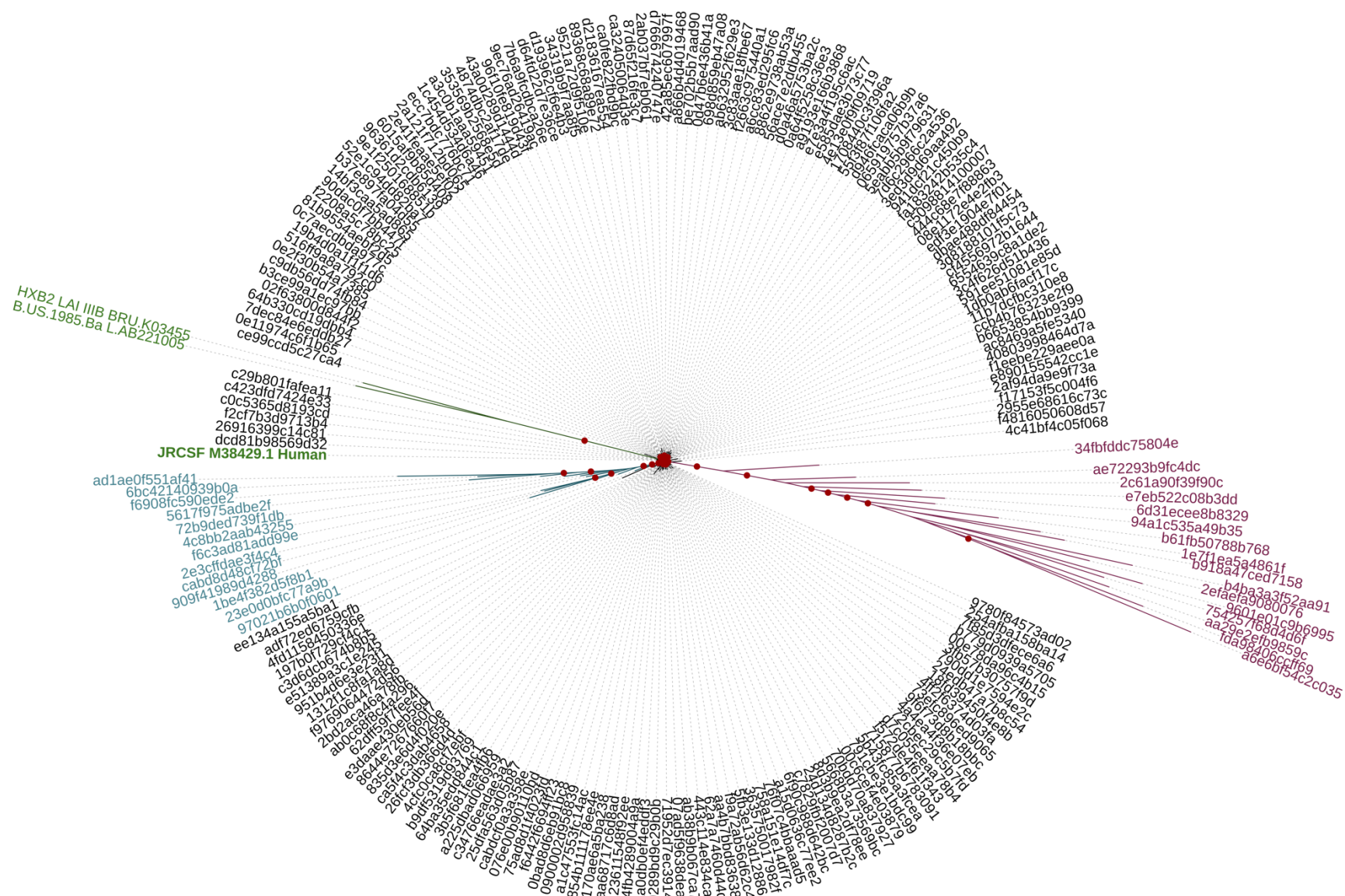

Supplement: S2 Fig — Phylogenetic analyses of recovered proviral genomes from the spleen of a PGX mouse reconstituted with lymphocytes from a human viremic controller demonstrates three statistically distinct populations. The majority of sequences (black) are very similar whereas the statistically significant divergence of two smaller outgroups (blue and pink, respectively) was supported by two independent bootstrap analyses methods (ultrafast and BOOSTER), as well as compartmentalization analysis, which achieved an AI = 0.00575, confirming robust population separation. (PDF) [file ppat.1013171.s002.pdf]
