# Supplementary material for: Development and validation of HIV SMRTcap for the characterization of HIV-1 reservoirs across tissues and subtypes
Source: PLoS Pathog. 2026 Jan 13;22(1):e1013171. doi: 10.1371/journal.ppat.1013171 (PMC12851485; doi:10.1371/journal.ppat.1013171)
Supplement: S2 Table — This table includes all viral load measurements taken from the viremic controller used in the PDX mouse model, highlighted in Fig 4 and S1 Table. (DOCX) [file ppat.1013171.s004.docx]

| **Supplementary Table 2. Complete Viral Load History of Viremic Controller used in PDX model** | |
| --- | --- |
|  |  |
| **Viral Load** | **Date** |
| <20 | 27MAR2018 |
| <40 | 25JAN2018 |
| <20 | 08NOV2017 |
| <20 | 02AUG2017 |
| <20 | 13APR2017 |
| <40 | 01FEB2017 |
| <20 (HIV-1 RNA detected) | 09JAN2017 |
| <40 | 12AUG2016 |
| <40 | 17MAY2016 |
| <40 | 19FEB2016 |
| <40 | 16NOV2015 |
| <40 | 24AUG2015 |
| <40 | 19MAY2015 |
| <40 | 09APR2015 |
| 80 | 11MAR2015 |
| <40 (not on ART) | 18DEC2014 |
| 480 | 17NOV2014 |
| 42 | 21MAY2014 |
| 59 | 25SEP2013 |
| 155 | 15MAY2013 |
| 1037 | 18DEC2012 |
