# Supplementary material for: Development and validation of HIV SMRTcap for the characterization of HIV-1 reservoirs across tissues and subtypes
Source: PLoS Pathog. 2026 Jan 13;22(1):e1013171. doi: 10.1371/journal.ppat.1013171 (PMC12851485; doi:10.1371/journal.ppat.1013171)
Supplement: Attachment — Submitted filename: Review of Sadri and Nadakal et al HIV SMRTcap.docx [file ppat.1013171.s005.docx]

Summary:

This well-written manuscript describes a novel method developed by Sadri and Nadakal et al., HIV Single Molecule Real Time Capture or HIV SMRTcap, for detection of intact HIV proviral genomes in conjunction with integration site analysis. This method is an important addition to HIV reservoir analysis tools and particularly valuable for its combined analysis of proviral integrity and integration site. HIV SMRTcap effectively utilizes long-read technology of Pacific Biosciences as well as cross-subtype probes to enrich HIV sequences in sheared DNA pools in a subtype-agnostic manner. The method is amenable to use in PBMCs and tissues from humans and tissues from humanized mice, and the authors have empirically compared HIV SMRTcap to other existing integration site and provirus analysis methods. HIV SMRTcap has the potential for high-throughput use and broad applicability to many sample types, and the methods have been well-described for reproduction by other groups. However, the manuscript would be improved with additional investigation to further define HIV SMRTcap’s sensitivity for intact HIV genomes and with other edits as detailed below.

Major Modifications/New Experiments:

1. HIV SMRTcap’s application to patient samples is perhaps the most important for the method’s value to the field. Although the samples in **Figure 7B-7C** were from patients undergoing long-term ART, it is unlikely the reservoirs of both these individuals harbor no intact proviral genomes, and they exist at a level that is simply below the limit of detection in this assay. Determining how the genomic-based HIV SMRTcap method compares to a functional assay such as QVOA for detection of replication competent provirus is essential for complete characterization of this new method, even though it is a semi-quantitative rather than fully quantitative method. Given that genomic-based assays typically overestimate intact provirus relative to functional assays, but HIV SMRTcap gives a lower proportion of intact genomes relative to IPDA, it will be very important to determine the relationship of SMRTcap to a functional assessment such as QVOA.
2. Although the titration of HIV genomes from 8e5 cells into the uninfected parent cell line was done with technical replicate libraries to determine limit of detection (**Figure 5**), the impact of different library preps with independent DNA shearing for the same sample on the frequency of intact HIV proviruses recovered was not addressed empirically. This is an important additional analysis to include regarding the reproducibility of the data across different libraries.

Minor Modifications/Editorial Suggestions:

1. I wasn’t able to find the supplementary material (FASTA files and .xlsx with supplementary table). This very well could be an error on my end, but please ensure that these relevant materials are included with the manuscript.
2. It appears from **Figure 1** and **Table 3** that circular consensus sequence (CCS) reads were used from PacBio, which is helpful to correct for the typically high sequencing error rate of this NGS method. Please ensure that the use of CCS rather than subreads is detailed in the Methods. Additionally, even with the use of CCS reads, PacBio has a notable tendency for erroneous single bp INDELs in polynucleotide tracts, which appear evident in the sequence alignment in **Figure 1**, particularly in the sequences that are called as PCR duplicates but are not 100% identical. How were INDELs and mutations that result from sequencing error handled in this analysis pipeline? These likely lowered the nucleotide % identity between both PCR duplicates and the shared regions of expanded clones to less than 100%; was there a minimum cutoff at which the identity was considered too low for sequences to be duplicates or expanded clones? Similarly, how did likely erroneous INDELs impact calls for intact proviruses in the pipeline?
3. There appear to be quite disparate results between this method and both IPDA and FLIP-Seq for intact provirus measures and LM-PCR for integration site analysis. Although some of the possible reasons for this are noted in the Results, please further describe and explore the potential causes for these disparities in the Discussion, and include a comparison of this method to MIPseq. Also, IPDA is described as a stimulation-based assay in the Discussion, which is incorrect.
4. **Figure 5A** is a little confusing for the scale of DNA mass in which 1815 is above 2062. Also, please label the horizontal line as 1 ug or simply omit the line and label the DNA mass axis in its units of ng. The calculation of HIV copies for each dilution in the manuscript text on page 12 is puzzling to me, as the 10,000 copies per 2 ug sheared DNA is given as 1 copy per 10^7^ genomes, and the additional 10-fold dilutions of HIV should then be 1 copy in 10^8^, 10^9^, and 10^10^ genomes. The text, however, gives 1x10^5^-1x10^8^ as the range of copies per host genome fragments. Please clarify the calculations and range for the HIV copies per host genome fragments, as the detection limit/sensitivity is an important component of the assay’s properties.
5. In **Figure 3C**, *gag* but not *nef* is shown as significantly different in the proportions of deletions in the subtype A vs. non-A viruses. This does not match the text on pages 9-10 that describes significant differences in *nef* as well as other accessory genes. Please clarify the genes that are significantly different in deletions between subtypes, and please align the manuscript text and figure legends for **Figure 3C** and **Figure 7D** regarding the level of gene segment that is intact (legend 0, 0-75 and text 0, 1-75). Additionally, it appears there might be a higher proportion across subtypes of proviruses with intact accessory genes *vif*, *vpr*, and *vpu* than intact structural genes *gag*, *pol*, and *env* (while *nef* appears more similar to structural genes) in **Figure 3C**. *Vif*, *vpr*, and *vpu* are considerably shorter than the structural genes; is the probability of detecting a gene/gene fragment different for the different genes? Are longer genes are more likely to be pulled out by the probes used in this method, or are probes in appropriate density across the genome to have equal likelihood of detecting any particular gene?
6. For the reference genomes utilized for probe design, how was subtype determined? It is possible that for each full-length genome included in the panel, if initially subtyped with only a specific gene, the full-length genome represents a recombinant rather than a pure subtype. Also, the GenBank Accession number for SE365 in **Table 1** appears incorrect and perhaps should be AB485649.
7. For the volunteer who passed of congestive heart failure and had a high level of intact HIV provirus in the heart (**Figure 4B**), are PBMCs or perhaps lymph nodes (heart-draining and non-heart draining) from this individual available to analyze for proviral compartmentalization and intact proviruses? Determining whether proviruses were a compartmentalized population in the heart or admixed with PBMCs/draining LNs would help support or refute this hypothesis of T cell infiltration due to inflammation in the heart.
8. Human cells for the mouse xenograft were provided by a donor described as an elite controller, yet he was receiving ART at the time of sample provision. Typically elite controllers are defined as such due to low VL without ART; please provide more information on the donor individual and his viral control history/ART history. The engrafted mice were also presumably monitored for infection by donor virus following engraftment and were uninfected prior to infection with JR-CSF. If so, please indicate in the Methods how this was done to demonstrate that the SMRTcap analysis was detecting proviruses from a single infection rather than superinfection by both donor and JR-CSF viruses. Please also provide the infection details for mice with JR-CSF (dose, route).
9. In the Circos plots for cell lines and human samples, please remove the Y chromosome if possible where it is not relevant for the sample type. (CEMs were collected from a female patient) This would better demonstrate in the figures that HIV provirus integrations weren’t detected in Y chromosomes not because they didn’t occur there, or occurred at a level below the limit of detection, but because the chromosome wasn’t present.
10. It’s not clear what cell lines were used for HIV enrichment across subtype experiments. SupT1/X4 and SupT1/R5 are given on page 8 then CEMs and SupT1s are described on page 9. SupT1/R5 and CEMs are in **Tables 2 and 3** but the Methods include SupT1/X4 and SupT1/R5 without any description of CEMs. Please clarify the cell lines used.
11. There are two typos on page 7 in the “Initial validation of HIV SMRTcap” section. **Table 2** should be **Table 3** in the second to last line of the page, and the third sentence of the section should read “…was also observed **in** multiple passages”. There is also a small typo on page 10 in the sixth line from the bottom: “(member of the National **NeuroHIV** Tissue Consortium)”.
12. In **Table 2**, brain tissue from the HIV negative donor is listed as both basal ganglia and frontal lobe. Please clarify the brain region for the sample.
13. Please provide an indication in the figure legend that the red box in **Figure 2A** is for the HIV genome. **Figure 2B** has the label for chromosome 4 obscured/missing, and a couple squares for LM-PCR appear to have snuck into the outer chord for SMRTcap analysis in **Figure 2C**. There is a missing mark for chromosome 2 in **Figure 4B**, and the chromosome 10 label is also missing in **Figure 6E**. Please also provide an explanation of the red circles in the phylogenetic tree for **Figure 7E** in the legend.
14. For author affiliations, 17 is given for an author before 16, and 4 and 9 appear to be the same institution. “TV” is also credited with providing primary samples, expertise, and editorial feedback while no author has these initials. Please edit the author attributions and affiliations.
15. Reference 16, “Cooperative action of CD8 T lymphocytes and natural killer cells controls tumour growth under conditions of restricted T-cell receptor diversity” by Shanker et al., does not seem relevant where cited. Please clarify the reason for citing.
