# Supplementary material for: Development and validation of HIV SMRTcap for the characterization of HIV-1 reservoirs across tissues and subtypes
Source: PLoS Pathog. 2026 Jan 13;22(1):e1013171. doi: 10.1371/journal.ppat.1013171 (PMC12851485; doi:10.1371/journal.ppat.1013171)
Supplement: Attachment — Submitted filename: HIV_SMRTcap_Methods_ReviewersCommentsOnly_FINAL_V2.docx [file ppat.1013171.s007.docx]

August 15, 2025

Dear Dr. Brenchley and our reviewers,

As corresponding author, I want to thank you and our reviewers on behalf of our team for very thorough and thoughtful comments in response to the submission of our manuscript, by Sadri, et.al., *Development and validation of HIV SMRTcap for the characterization of HIV-1 reservoirs across tissues and subtypes*. We have addressed all comments below in an itemized fashion, and we believe that the resulting, updated manuscript is much stronger after incorporating the constructive feedback. We hope that, after reviewing our revised manuscript, the team will find it acceptable for publication in PLoS Pathogens.

*Itemized responses to editorial and reviewer comments are below. For ease of interpretation, editorial and reviewer comments are italicized and our responses are in* standard text:

**Reviewers' Comments:**

In response to several reviewers’ comments, page and line numbers have been added to facilitate re-review and will be included in future submissions.

**Part II – Major Issues: Key Experiments Required for Acceptance**

Reviewer #1:

1. *It seems there is one integration site detected by LM-PCR on the Y-chromosome. Yet, the parental cell line to generate 8e5 cells was derived from a female with acute lymphoblastic leukemia. How to explain this finding? Could this represent a technical/analysis artifact that has also affected other results in the study?*

We thank Reviewer #1 for this observation and appreciate that it provided a very valuable “sanity check” in evaluating our data manually in preparation for this response. The first point we’d like to note is that this false positive integration site signal mapping to the Y chromosome in Figure 2C was identified in the linker-mediated PCR (LM-PCR) integration site profiling data only; this site was not identified or mapped as part of the HIV SMRTcap pipeline. When we examined the data further, it appeared that this integration site sits within highly repetitive genomic content and was likely mis-mapped due to the short-read nature of these data, which do not contain sufficient unique sequence content to more accurately ascertain where the integration site occurs. As this integration site is patently false due to the biological sex of the original donor, we have removed this signal from our circus plot to avoid reader confusion. With that noted, it does draw attention to potential artifactual data due to short-read mapping errors that may exist in the larger LM-PCR dataset. However, as the aim of this manuscript was the proof-of-concept demonstration of the HIV SMRTcap pipeline, and not detailed integration site pipeline comparisons, we have not further explored these possible artifacts with any type of systematic analyses.

2. *Figure 5B: The manuscript states “Robust HIV SMRTcap detection of >200 HIV templates was observed at the lower dilutions (e.g., containing more HIV templates) than expected.” This reviewer found this sentence to be confusing, and possibly inaccurate. Would it be fair to rewrite the sentence as “Robust HIV SMRTcap detection of >200 HIV templates was observed at lower dilutions than expected, suggesting greater sensitivity than expected.”? Also, the figure seems to suggest that more 8e5 cell DNA may have been loaded in the first dilution than intended, so that detecting HIV templates at the lower dilution does not necessarily reflect high assay sensitivity. Please clarify this.*

We appreciate this comment. It was clear, based on multiple reviewer comments, that the limit of detection experiments and results were not sufficiently explained in our first manuscript draft. This and the other related comments have improved the clarity of this subsection. The 8e5 dilutions characterized in Figure 5 with the aim of assessing HIV SMRTcap limits of detection represented both technical and biological replicates to additionally assess reproducibility. This ensured that results reflected multiple sample inputs, such that one mistake with dilutions (possibly suggested by the reviewer) would not result in artifactual results carried through the entire experiment. To ensure sufficient clarity regarding experimental design, we have extensive reworded the subsection entitled “HIV SMRTcap Limits of Detection” (starting at Line 302) and have restructured Figure 5 and its corresponding legend to include additional sample-specific input details. As part of this effort, we have now adopted the phrasing suggested by this reviewer.

3. *Figure 5C: HIV copies recovered with SMRTcap were higher in the 10 HIV input template dilutions than in the 100 HIV input template dilutions. How to explain this discrepancy?*

As described above, it was clear from our review comments that additional information was required to explain Figure 5 and the limit of detection experiments, we again thank the reviewers for encouraging us to improve our manuscript significantly in this area. To address this specific point, we anticipate that down-sampling of proviral templates at each stage of the HIV SMRTcap molecular protocol is likely semi-stochastic for several reasons: (i) while oligonucleotide enrichment increases the relative proportion of provirus-containing fragments, probe binding is competitive and up to ~30% of each enriched library is non-specific genomic content; and (ii) the input mass used to load the Revio system, particularly with the new SPRQ chemistry can be as low as 1 ng captured product library. Together this means that, even in the most minimal of scenarios, a single Revio cell will be flooded with over three times the absolute number of DNA fragments than can be loaded into each well (Zero-Mode Waveguides, 25 million per SMRTcell) and able to proceed with sequencing. This results in a non-specific sampling biasper sequencing reaction that can lead to the same inputs provided to sequencing, but variation in the actual molecules detected in the data itself. Particularly at low concentrations of the fragment of interest, as there was in the case of detecting a “signal” of 10-100 templates out of the ~75M DNA molecules loaded, the “noise” of non-specific DNA (mostly derived from the CEM HIV negative sample) enhanced variability of detection. This interpretation would be the same for the earlier data generated on the Sequel IIe, which only contained 8M wells, but needed to be loaded with 10 times the material.

There is also variation in the loading of the system(s), due to SMRTcell-to-SMRTcell variation. Given the varied platforms used and the possibility of variable loading, we have added a table to Figure 5 showing the total number of CCS reads for each sample. For example, replicate n3 was sequenced on the Sequel IIe, not the Revio system used for the other replicates. Although our data demonstrates that HIV SMRTcap can detect viral DNA at very low concentrations (as few as 10 HIV DNA copies) and thus suitable for studying HIV reservoirs in ART-suppressed individuals, we do not claim that HIV SMRTcap is a fully quantitative method, primarily due to these signal-to-noise issues at low viral concentrations.

Reviewer #2:

1. *HIV SMRTcap’s application to patient samples is perhaps the most important for the method’s value to the field. Although the samples in* ***Figure 7B-7C*** *were from patients undergoing long-term ART, it is unlikely the reservoirs of both these individuals harbor no intact proviral genomes, and they exist at a level that is simply below the limit of detection in this assay. Determining how the genomic-based HIV SMRTcap method compares to a functional assay such as QVOA for detection of replication competent provirus is essential for complete characterization of this new method, even though it is a semi-quantitative rather than fully quantitative method. Given that genomic-based assays typically overestimate intact provirus relative to functional assays, but HIV SMRTcap gives a lower proportion of intact genomes relative to IPDA, it will be very important to determine the relationship of SMRTcap to a functional assessment such as QVOA.*

We thank this reviewer for their kind words; we also concur that performance in patient samples is a critical proof-of-concept. Although this manuscript was not intended to be a comprehensive comparison across HIV reservoir characterization methods, we do acknowledge that the discrepancy between methods, particularly in identifying intact proviral genomes was surprising to us as well. Ultimately, we believe that the noted discrepancy in detection of intact proviral genomes between HIV SMRTcap and other assays may be a byproduct of limited primer binding site presence as a proxy measurement of intactness (as is the case in IPDA), or a the difference in starting material in which resting CD4 T cells are first sorted before reservoir characterization is performed likely enriching the overall frequency of HIV-containing cells examined (as in many QVOA approaches). We strongly believe that the ability to resolve intactness directly from the sequencing data, rather than the use of proxy measures, leads to more trustworthy “intact” calls from the data alone, which can be further validated with orthogonal methods. To address the observed disparities in the text, we’ve added the following text (line 469):

“The discrepancy in detection of “intact” proviruses between pre-existing methods and HIV SMRTcap likely represents the more stringent definition of intactness in HIV SMRTcap data, enabled by nucleotide-level resolution of integrated proviral content. However, a provirus may retain small deletions in accessory genes as observed here and be categorized as “internally deleted” by HIV SMRTcap but still maintain inducibility and replication competence *in vivo*. In such a situation, functional validation using assays such as the quantitative viral outgrowth assay (QVOA) could be used to evaluate the differences between “internally deleted” and truly “defective” proviral genomes.”

We also agree that comparison to functional reservoir data, such as QVOA, from the same samples could be informative. Luckily, several of the samples highlighted in Figure 6 were previously characterized with QVOA and this gave us the opportunity to incorporate this comparison without having to generate additional data. We have added the QVOA data for matched samples as a table-based panel in Figure 6 and updated the corresponding figure legend with the appropriate information. In these comparisons, the QVOA did identify a very low level of infectious virus, assumed to be intact, which still remains distinct from the HIV SMRTcap data. Here we believe that these very low levels of infectious genomes may be under the limit of detection of the HIV SMRTcap assay, as the QVOA used pre-sorted resting CD4+ T cells as input, whereas HIV SMRTcap for these samples used total PBMC. Further, infectivity does not necessarily require completely intact proviral genomes; thus, infectious units, as measured by QVOA, may still contain small deletions in accessory genes, which would be detected as internal deletions in HIV SMRTcap, but still result in replication competent viruses. We noted this in the text as well, stating (line 476):

“In fact, parallel evaluation of samples by HIV SMRTcap and QVOA did indicate that replication-competent, inducible proviruses were present in these individuals at the time points indicated in Figure 6D. However, the frequency of these infectious units was extremely low (< 1 IUPM), and may not have been represented as intact genomes in the HIV SMRTcap data because either (i) they were under the limit of detection for HIV SMRTcap, which was performed using total PBMC gDNA input, compared to pre-sorted resting CD4+ T cells in QVOA; or (ii) these replication-competent proviruses still contained small deletions in accessory genes and may be represented within the HIV SMRTcap data, but these deletions did not impact inducibility or infectivity. These possibilities underscore the importance of functional, orthogonal validation of all genomics-based HIV reservoir characterization methods.”

2. *Although the titration of HIV genomes from 8e5 cells into the uninfected parent cell line was done with technical replicate libraries to determine limit of detection (****Figure 5****), the impact of different library preps with independent DNA shearing for the same sample on the frequency of intact HIV proviruses recovered was not addressed empirically. This is an important additional analysis to include regarding the reproducibility of the data across different libraries.*

We thank the reviewers for this valuable feedback. The data presented in Figures 5A and 5B represent both biological and technical replicates, incorporating different cell batches, independent library preparations, and separate sequencing runs. We realize now that we did not highlight this appropriately with the original wording. We have revised the text in the “HIV SMRTcap Limit of Detection” subsection to more clearly describe the nature of these replicates and have added details specifying the type of replicate for each sample in Figure 5B.

Reviewer #3: (No Major Issues)

**Part III – Minor Issues: Editorial and Data Presentation Modifications**
Please use this section for editorial suggestions as well as relatively minor modifications of existing data that would enhance clarity.

Reviewer #1:

1. *Table 1 shows the sequences used to design HIV specific probes, but does not indicate probe sequences or target genes. Please include a table indicating sequences and targets for each probe.*

We appreciate the reviewer’s comment, but unfortunately, the probe sequences are proprietary information (patent pending) and therefore cannot be shared publicly.  However, the probe mix is available for use by other groups upon request to the corresponding author.

2. *This reviewer could not find Supplementary Table 1.*

We sincerely apologize for this oversight, as we had attached tables as separate documents during our original submission. We have incorporated all tables into the main manuscript text document and the Supplementary data and tables are also in a combined document for our revised submission.

3. *Figure 2C: there are two open square symbols displayed on the outer chord chr 2 - are these integration sites displayed on the wrong chord?*

We thank Reviewer #1 for catching this discrepancy, which was an artifact of merging several versions of the figure-generating code. We have updated the script and produced an updated Figure 2C, which does plot the correct value. Per Reviewer #2, Minor Revision #9, the Y chromosome was also removed for clarity.

4. *Figure 3C: Text in the manuscript indicates that “93-RW/subtype A showed significantly more small deletions of HIV accessory genes vif, vpr, vpu, and nef than the other viruses,” yet the figure shows that deletion of nef in the subtype A virus was not significantly different compared to other subtypes. Please clarify this text.*

This is an oversight that we appreciate was noticed by Reviewer #1. To ensure clarity, we edited the following text starting at line 246:“The relative pattern of proviral internal deletions did appear different in 93-RW (HIV-1 subtype A) versus subtypes B, C and D. Notably, 93-RW/subtype A showed more extensive small deletions of HIV-1 accessory genes *vif, vpr, and vpu* alongside a notable difference in structural gene *gag,* than the other viruses.” These edits also reflect points made by Reviewer #2, Minor Revision #5.

5. *Figure 4: Colors indicating internal deletion or truncated proviruses are difficult to distinguish on the chord diagram, please consider changing color selections.*

All categorical colors were changed to improve contrast as requested.

6. *Figure 5A: This reviewer was unclear on what the horizontal dashed line represents.*

The horizontal line indicates 1000 ng of DNA. This has been clarified in the Figure 5 legend. We thank the reviewer for this note to make Figure 5 clearer to the reader.

7. *Figure 6: How many PBMC were processed to characterize the reservoir in ART-treated participants P4, Donor 17 and Donor 13? For Donor 17 and Donor 13? Are the differences in the number of proviruses recovered in each timepoint related to the input of cells for sequencing?*

For each sample, we extract genomic DNA from an average of 5 million cells (range 2.2M - 8.5M). To normalize input to facilitate sample to sample comparison, we sheared only 2.5 µg of the extracted gDNA and use 1 µg of this sheared gDNA as assay input. By using the same initial sample type, this standard input should provide some level of normalization as to the number of cell equivalents used (although we do not track distribution of leukocyte types in each PBMC sample, which can and does differ per individual). Assuming that each human cell contains approximately 6 pg of gDNA, this 1 µg corresponds to ~166,000 cell used for input. The standardized amount of input DNA should limit concerns about variable results as a factor of input cell number; however, it could reflect different cell-type distributions per individual, actual viral burden in each sample, and/or the inherent sampling bias associated with low-abundance targets (discussed above regarding Figure 5 results).

8. *Figure 6B: Two clones are shown integrated in chromosome 17 at positions only 5 bases apart (40646538 and 40646543). The manuscript suggests this likely represents the same clone captured from either the 5' or 3' end of the integrated provirus. Please clarify whether a similar situation applies to the two clones integrated in chromosome 5. Additionally, the boxes indicating integration sites currently overlap with the pie chart, obscuring the dots representing individual proviruses. It would be best to reposition these boxes so that all data points remain visible.*

We sincerely thank the reviewer for catching this analysis artifact. We reviewed these data manually and, indeed, as suggested, the noted integrations in chromosome 5 are the same. To address the formatting comment, we have regenerated the figure and repositioned the boxes as specified so all data points remain visible.

9. *Figure 6: The plots from all donors show that there are some clones with the same integration site, but it is unclear if these are the same clone sheared in different locations or if instead they are different clones that differ by a few bases. Please include a table with all information of the integration sites (Ex: chromosome, location, and provirus orientation).*

We appreciate this feedback and believe it represents the need for improved textual clarity within the manuscript. We designated integration sites as the same clone if their integration coordinates overlapped. For these clones, each was then re-assigned coordinates based on the outer bounds of each overlapping integration event. Future work will incorporate sequence identity to further refine clonal identity within close proximity. To clarify this, we added this additional text at line 697: “Similarly, integration coordinates between different reads that overlapped (e.g. chr1:300,600,428-300,600,432 and chr1:300,600,430-300,600,433) were considered clones of the same initial HIV integration event.” The integration site information provided is present in the mastersheet deliverable for each sample upon completion with our HIV SMRTcap analysis pipeline. Supplementary Table 1 is an example of this document for the PGX mouse model, for which the Circos plot providing an overview of integration is shown in Figure 4C.

Reviewer #2:

1. *I wasn’t able to find the supplementary material (FASTA files and .xlsx with supplementary table). This very well could be an error on my end, but please ensure that these relevant materials are included with the manuscript.*

As noted in response to Reviewer #1, Minor Revision #2, this was a mistake made during submission. We have now incorporated the missing supplementary data into a single document that is visible on our end. We apologize for any confusion.

2. *It appears from* ***Figure 1*** *and* ***Table 3*** *that circular consensus sequence (CCS) reads were used from PacBio, which is helpful to correct for the typically high sequencing error rate of this NGS method. Please ensure that the use of CCS rather than subreads is detailed in the Methods. Additionally, even with the use of CCS reads, PacBio has a notable tendency for erroneous single bp INDELs in polynucleotide tracts, which appear evident in the sequence alignment in* ***Figure 1****, particularly in the sequences that are called as PCR duplicates but are not 100% identical. How were INDELs and mutations that result from sequencing error handled in this analysis pipeline? These likely lowered the nucleotide % identity between both PCR duplicates and the shared regions of expanded clones to less than 100%; was there a minimum cutoff at which the identity was considered too low for sequences to be duplicates or expanded clones? Similarly, how did likely erroneous INDELs impact calls for intact proviruses in the pipeline?*

We thank Reviewer #2 for this feedback, as understanding the accuracy of the data we present is a critical feature of the HIV SMRTcap pipeline. In response, we have added the following detail to the Methods section to clarify the read types generated by the Sequel IIe and Revio systems, and how these reads were quality filtered for use in the HIV SMRTcap analyses downstream (line 664): “CCS reads at a Phred Quality score of 30, or 99.9% accurate, or higher were determined to be High Fidelity (HiFi) reads.”

All analyses used high fidelity (“HiFi”) circular consensus (CCS) reads, which are defined as CCS reads ≥ Q30, or 99.9% accurate. The average accuracy of the HIV SMRTcap data presented in this study, summarized in Table X, was Q38 (99.98% accurate). Thus, we anticipate two nucleotide-level errors per 10,000bp, which is the average size of our sequenced insert(s). We do not anticipate these errors to impact our ability to identify clones, as we use the integration site coordinates in combination with the flanking regions to identify clonal populations. To account for possible polymerase slippage during the two PCR steps used for enrichment, we allow for a ± 5bp window on either side of a common integration site to be grouped within a clonal lineage. In terms of identifying intact proviral genomes, we provide a wide berth of divergence, defining “intact” if gene identity to viruses of the same subtype is ≥75%. This allows for intrasubtype diversity, which can be greater than 20% within subtypes within highly divergent genes (e.g. *env*) to result in erroneous integrity calls.

3. *There appear to be quite disparate results between this method and both IPDA and FLIP-Seq for intact provirus measures and LM-PCR for integration site analysis. Although some of the possible reasons for this are noted in the Results, please further describe and explore the potential causes for these disparities in the Discussion and include a comparison of this method to MIPseq. Also, IPDA is described as a stimulation-based assay in the Discussion, which is incorrect.*

We thank the reviewer for this helpful comment, which has led us to have several in-depth discussions regarding the many HIV reservoir characterization methods and their respective pros and cons. However, we did not intend this paper to be a comprehensive comparison across methods and, therefore, intentionally have minimized comparative discussions. We believe that each method has its own benefits and limitations, which are relatively unique to the strategy chosen by the investigator as the best fit for the scientific question at hand. Moreover, in many ways, HIV SMRTcap can complement these orthogonal methods by offering added information (e.g., proviral integrity information to an integration site-only approach) or acting as a high level screen of bulk repertoires that can be further dissected using single genome amplification approaches (e.g., FLIP-seq or MIP-seq) once reservoirs of interest are chosen. As our focus was not on complete head-to-head comparison of all methods, we be unable to perform MIP-seq on these samples due to sample limitations, cost, and time restrictions.

Regarding the typo labeling IPDA as a stimulation-based assay, that text was removed.

4. ***Figure 5A*** *is a little confusing for the scale of DNA mass in which 1815 is above 2062. Also, please label the horizontal line as 1 ug or simply omit the line and label the DNA mass axis in its units of ng. The calculation of HIV copies for each dilution in the manuscript text on page 12 is puzzling to me, as the 10,000 copies per 2 ug sheared DNA is given as 1 copy per 10^7^ genomes, and the additional 10-fold dilutions of HIV should then be 1 copy in 10^8^, 10^9^, and 10^10^ genomes. The text, however, gives 1x10^5^-1x10^8^ as the range of copies per host genome fragments. Please clarify the calculations and range for the HIV copies per host genome fragments, as the detection limit/sensitivity is an important component of the assay’s properties.*

We thank this reviewer for highlighting an area of particular confusion for all reviewers and, further, for providing concrete points for which clarification was needed. To do so, much of the “Limits of Detection” subsection has been rewritten to improve clarity and understanding (starting on line 307). Briefly, as the oligo-based capture happens on gDNA fragments that are ~10kb in length, and not at the intact chromosome level, we have calculated and reported assay sensitivity as the HIV genome fragment(s) per 10kb gDNA fragments, and all limits of detection values have been reported as DNA fragments, not copy number, to avoid confusion. Additionally, Figure 5A and its corresponding legend have been edited accordingly.

5. *In* ***Figure 3C****, gag but not nef is shown as significantly different in the proportions of deletions in the subtype A vs. non-A viruses. This does not match the text on pages 9-10 that describes significant differences in nef as well as other accessory genes. Please clarify the genes that are significantly different in deletions between subtypes, and please align the manuscript text and figure legends for* ***Figure 3C*** *and* ***Figure 7D*** *regarding the level of gene segment that is intact (legend 0, 0-75 and text 0, 1-75). Additionally, it appears there might be a higher proportion across subtypes of proviruses with intact accessory genes vif, vpr, and vpu than intact structural genes gag, pol, and env (while nef appears more similar to structural genes) in* ***Figure 3C****. Vif, vpr, and vpu are considerably shorter than the structural genes; is the probability of detecting a gene/gene fragment different for the different genes? Are longer genes are more likely to be pulled out by the probes used in this method, or are probes in appropriate density across the genome to have equal likelihood of detecting any particular gene?*

We thank the reviewers for their diligent attention to detail, which has done much to improve our study. To address this comment, we have added the following sentence (line 246): “The relative pattern of proviral internal deletions did appear different with 93-RW (HIV-1 subtype A) versus subtypes B, C and D. Notably, 93-RW/subtype A showed more extensive small deletions of HIV-1 accessory genes *vif, vpr, and vpu* alongside a notable difference in structural gene *gag,* than the other viruses.” This line does better reflect the accessory gene data. Further, the bounds of each intactness category have been similarly amended to be concordant with Figure Legends 3C and 7D.

Regarding differential probability of detecting genes, the enrichment is performed on a long, single molecule based on tiling of 120-mer HIV-specific oligonucleotides across the region. Only 25% of the HIV sequence (~2500bp) contained within needs to be bound to probe to capture the entire fragment, these probes can be bound non-consecutively (e.g. 25 120-bp probes bound randomly throughout the span of the proviral molecule). So, for example, it would be possible to enrich a full-length proviral genome with no probes binding in *env*. So, in this case, we do not anticipate shorter genes being at a disadvantage during capture, as the entirety of the molecule is captured intact.

6. *For the reference genomes utilized for probe design, how was subtype determined? It is possible that for each full-length genome included in the panel, if initially subtyped with only a specific gene, the full-length genome represents a recombinant rather than a pure subtype. Also, the GenBank Accession number for SE365 in* ***Table 1*** *appears incorrect and perhaps should be AB485649.*

We thank Reviewer #2 for providing the opportunity to clarify the references used for our panel design. Subtype-specific reference genomes were obtained from the Los Alamos HIV Sequence Database (<https://www.hiv.lanl.gov/content/sequence/NEWALIGN/align.html>) , and were all retrieved as full-length viral genomes, not as individual genes, and subtypes were stated in the paper as presented by LANL.

Regarding the second point, we have changed the SE365 GenBank accession number as highlighted and thank the reviewer for catching this typographical error.

7. *For the volunteer who passed of congestive heart failure and had a high level of intact HIV provirus in the heart (****Figure 4B****), are PBMCs or perhaps lymph nodes (heart-draining and non-heart draining) from this individual available to analyze for proviral compartmentalization and intact proviruses? Determining whether proviruses were a compartmentalized population in the heart or admixed with PBMCs/draining LNs would help support or refute this hypothesis of T cell infiltration due to inflammation in the heart.*

We agree with the reviewer that this case is particularly interesting! Unfortunately, at the time of experimental design, lymph node samples were unavailable and PBMC have not been stored. We are discussing the possibility of following up on these observations with a more dedicated anatomical atlas-type case study of this individual, but that would be out of scope of this methods-focused manuscript.

8. *Human cells for the mouse xenograft were provided by a donor described as an elite controller, yet he was receiving ART at the time of sample provision. Typically elite controllers are defined as such due to low VL without ART; please provide more information on the donor individual and his viral control history/ART history. The engrafted mice were also presumably monitored for infection by donor virus following engraftment and were uninfected prior to infection with JR-CSF. If so, please indicate in the Methods how this was done to demonstrate that the SMRTcap analysis was detecting proviruses from a single infection rather than superinfection by both donor and JR-CSF viruses. Please also provide the infection details for mice with JR-CSF (dose, route).*

We appreciate the reviewer’s comment. To follow up, we obtained additional clinical data for this donor and have updated the manuscript to more accurately describe the donor as a viremic controller (line 292). The infectious dose of the donor virus has been updated in Table 1. Viral load history for this individual is now reflected as Supplementary Table 2, incorporated into the main mansucript. We additionally investigated whether the viruses recovered with HIV SMRTcap derived only from the clonal JR-CSF, or if we could detect divergent donor viruses. Now represented in Supplementary Figure 2, two statistically significant outgroups of virus were detected that may represent host-derived virus, corresponding text on Line 299. Importantly, SMRTcap is designed to detect HIV proviruses regardless of their source.

9. *In the Circos plots for cell lines and human samples, please remove the Y chromosome if possible where it is not relevant for the sample type. (CEMs were collected from a female patient) This would better demonstrate in the figures that HIV provirus integrations weren’t detected in Y chromosomes not because they didn’t occur there, or occurred at a level below the limit of detection, but because the chromosome wasn’t present.*

We thank the reviewer for this observation; Y chromosomes have been removed from Figures 2B, 2C, 6C, and 6E as suggested and as aligns with the biological sex of the sample.

10. *It’s not clear what cell lines were used for HIV enrichment across subtype experiments. SupT1/X4 and SupT1/R5 are given on page 8 then CEMs and SupT1s are described on page 9. SupT1/R5 and CEMs are in* ***Tables 2 and 3*** *but the Methods include SupT1/X4 and SupT1/R5 without any description of CEMs. Please clarify the cell lines used.*

We thank the reviewer for this observation, correction of which will certainly help with reader clarity. The necessary tables and text have been updated to clarify which cell lines were used in which experiment as negative control(s).

11. *There are two typos on page 7 in the “Initial validation of HIV SMRTcap” section.* ***Table 2*** *should be* ***Table 3*** *in the second to last line of the page, and the third sentence of the section should read “…was also observed* ***in*** *multiple passages”. There is also a small typo on page 10 in the sixth line from the bottom: “(member of the National* ***NeuroHIV*** *Tissue Consortium)”.*

Typos have been corrected in the text. Thank you for catching them.

12. *In* ***Table 2****, brain tissue from the HIV negative donor is listed as both basal ganglia and frontal lobe. Please clarify the brain region for the sample.*

The tissue used as the negative control was collected from the frontal lobe. Edits have been made to Table 2 accordingly.

13. *Please provide an indication in the figure legend that the red box in* ***Figure 2A*** *is for the HIV genome.* ***Figure 2B*** *has the label for chromosome 4 obscured/missing, and a couple squares for LM-PCR appear to have snuck into the outer chord for SMRTcap analysis in* ***Figure 2C****. There is a missing mark for chromosome 2 in* ***Figure 4B****, and the chromosome 10 label is also missing in* ***Figure 6E****. Please also provide an explanation of the red circles in the phylogenetic tree for* ***Figure 7E*** *in the legend.*

Thank you for noting several typographical errors in the text that interfere with reader clarity. To address these: (i) we have added the following sentence to the description of subfigure A in the legend of Figure 2 at line 1030: “Proviral sequence is denoted by the genomic content contained within the red box.”; (ii) the figures identified in this comment have been modified to remove the noted overlaps and missing labels; (iii) corrections to Figure 2C have been made; and (iv) the red dots in Figure 7E indicate bootstrap values >0.9 for significant cluster separation; these details have been to the Figure 7 legend.

14. *For author affiliations, 17 is given for an author before 16, and 4 and 9 appear to be the same institution. “TV” is also credited with providing primary samples, expertise, and editorial feedback while no author has these initials. Please edit the author attributions and affiliations.*

We have re-numbered author affiliations, removed redundancies, and edited the typo “TN” to the correct “TK”.

15. *Reference 16, “Cooperative action of CD8 T lymphocytes and natural killer cells controls tumour growth under conditions of restricted T-cell receptor diversity” by Shanker et al., does not seem relevant where cited. Please clarify the reason for citing.*

We thank the reviewer for their extremely close reading of the test. This was an erroneous citation and has been removed.

Reviewer #3:

1. *A key conclusion of the paper is that HIV SMRTcap provides a more detailed view of proviral integrity than qPCR-based methods like the Intact Proviral DNA Assay (IPDA): SMRTcap identifies accessory gene deletions that IPDA would miss (Fig 7B-D). While technically true, the discussion of this point requires more nuance. The IPDA is designed to quantify proviruses that are likely replication-competent, using probes in gag and env as a proxy. HIV SMRTcap, by contrast, defines "intactness" bioinformatically as having >75% of all 11 viral genes present. It is not clear that the accessory gene deletions identified exclusively by SMRTcap would render a provirus replication-incompetent. Therefore, calling these proviruses "defective" in the same category as those with major structural gene deletions might be an overstatement. The authors should discuss this distinction more carefully. Are these assays measuring different things (potential for replication vs. genomic completeness), and if so, how should they be used together? The authors are probably right, but neither approach is the “gold standard”, i.e., actual replication assays. The 75% threshold for calling a gene "intact" feels somewhat arbitrary. Some justification for how this cutoff was determined and validated would strengthen the integrity classification schema. How sensitive are the results to this specific value?*

We appreciate the thoughtful comments made by Reviewer #3, which highlight the very true differences between our method and IPDA. As specified above, we did not aim to perform a comprehensive methods comparison of HIV SMRTcap to the suite of reservoir characterization tools available, but only to place our novel method in context with commonly used existing methods. We agree and have stated that our method likely would work well in concert or as an orthogonal validation of others. Further, as intrasubtype nucleotide-level variation can be present at rates ranging from 20-30%, the 75% identity threshold was chosen to avoid placing too stringent of a cut-off, such that natural within-subtype variation would be misclassified as a internal deletion. We evaluated several thresholds during the pipeline optimization process, and 75% identity best matched ground truth when manually curated. Higher values led to increased false negative calls; while lower values led to increased false-positive calls regarding the presence/absence of the majority of necessary gene content for function. We have added these clarifying details in the Materials and Methods.

The request of more nuanced discussion of IPDA is noted, and the text has been edited accordingly (starting at Line 415): “Despite this, IPDA is not used to explicitly capture and characterize proviral intactness but to quantify proviruses that are likely to be replication competent. While the noted HIV SMRTcap-detected deletions in accessory genes may not render a virus replication-incompetent or completely defective in the same way that deletions in *gag* or *env* may, altered accessory gene expression can directly impact viral pathogenesis by altering susceptibility to antiviral factors key to immune system activity.” That said, the use of the term “defective” was chosen to agree with existing norms regarding internal deletions in HIV.

2. *The limit of detection experiment is useful and informative, but the results presented in Figure 5 raise questions. The authors show that at very low dilutions, the empirically detected number of HIV templates exceeds the number predicted by their subsampling model. This is an unexpected and interesting result.
   Could the authors speculate on why this might be? Does the model, which is based on technical replicates, not fully capture the dynamics of the assay? Could there be more efficient hybridization or capture when the target is extremely rare? A more thorough discussion of this discrepancy would be beneficial. The variability in detection at the lowest dilution (2 of 4 replicates) should also be acknowledged more directly in the discussion of the assay’s sensitivity. While impressive, users need to be aware of the stochastic nature of detection at such low frequencies.*

We appreciate this comment on our Limit of Detection subsection, which (as noted above) has undergone extensive editing to enhance overall reader clarity. We firmly agree with Reviewer #3 that the observed results at very low template concentrations likely stems from sampling bias, and, further, would be influenced by differences in sequencing depth. We have additionally highlighted these variables in Figure 5 by displaying the total number of CCS reads resolved for each sample. We do not believe the assay is more sensitive at extremely low target levels, as the higher dilution still represents an extremely rare event—estimated at approximately 1 HIV copy per 10¹⁰ 10 kb fragments of the human genome. While SMRTcap may be sufficiently sensitive to capture HIV at these low frequencies, it is not intended to function as a quantitative assay especially under such conditions, where the influence of sampling bias becomes substantial.

3. *The use of unique shearing-site “endogenous barcodes” to distinguish PCR duplicates from true clonal expansion is clever. However, the operational definition of an identical clone—integration sites falling within a 10 bp window—could be problematic. While intended to account for polymerase slippage, it also risks conflating distinct integration events in genomic “hotspots” into a single clone. Providing a reference or further justification for this 10 bp window would be helpful. Furthermore, the authors correctly identify a current limitation in Figure 6, where 5’ and 3’ single-flanked reads from what could be the same clone are analyzed separately. Because could lead to an underestimation of clone size and an overestimation of clonal diversity, this limitation should be more prominently stated in the Results and Discussion sections, rather than being presented primarily as a future pipeline development.*

We thank Reviewer 3 for this thoughtful feedback and agree about the possible risk of conflating distinct integration events in extremely close proximity and polymerase slippage. To address this, we edited our definition of our 10bp window, and provided further references justifying our selection, at line 694: “10 bp was chosen as the maximal spread with which the 5’ and 3’ integration sites could differ and still be called as the same clone due to well-described polymerase slippage of up to 5 bp on either side of the integration site, and existing technical limitations of all sequencing platforms with homopolymeric sequence identity (71, 72).”

In regard to the possibility of underestimating clone size and overestimating clonal diversity, we added text to the Discussion to clarify this limitation of HIV SMRTcap (line 460): “If resolved as only a single-flanked (5’ or 3’ only) provirus due to shearing artifacts or only as a single template with this integration site, this could lead to erroneous clonal identification, functional underestimation of clone size, exaggeration of clonal diversity, or hinderance in identifying local integration hotspots.”

4. *In the clonality pie charts (Fig 6D, 6F), it is difficult to track the clinically relevant clones mentioned in the text (e.g., in CCR3 or BACH2) without any labels. Annotating the largest or most persistent clones directly on the charts or in a corresponding table would greatly aid interpretation.*

We thank the Reviewer for all comments aimed at enhancing reader clarity given the novel nature of our data representations. We have added the requested labels and agree it helps follow our narrative.

5. *The circos plot in Figure 2B contains a lot of information (genic/intergenic, repeat/non-repeat). Distinguishing between the triangles and circles, and open versus filled symbols, is difficult at a glance. These figures (and some others) also violate the cardinal Tufte data viz rule, by having low data to ink ratio (a lot of wasted white space for example).*

We appreciate the concerns described by Reviewer #3. Given the novelty of the data – linking human integration site data with proviral integrity, the circos plot format was used to summarize a large amount of data at a high level. While Figure 2B is relatively sparse because of its use as a benchmarking figure, we believe it is needed to convey identification of 8e5 integration sites beyond the canonical chromosome 13 site, setting the stage for the use of circos plots in future figures summarizing primary sample data.

6. *In the discussion, the authors note that HIV SMRTcap likely fails to capture hypermutated proviruses, a known limitation of hybridization-based methods. They reasonably argue this is "not considered a critical fault" since such viruses are _probably_ not replication competent. However, for studies aiming to characterize the entire landscape of proviral DNA, this is a notable bias that should be clearly and strongly stated.*

We agree with Reviewer #3’s concern as to this limitation of the HIV SMRTcap pipeline. We have added text (line 463) to elaborate on the limitations associated with capturing hypermutated proviruses: “HIV SMRTcap is likely also limited in its ability to capture highly hypermutated proviruses, as the extensive sequence divergence would be expected to reduce overall capture efficiency and recovery of these variants.”

7. *The discussion claims the method is "cost-effective." While this may be true, the manuscript provides no explicit data to support this. I would suggest either removing this statement or softening it (e.g., "HIV SMRTcap may reduce overall costs by consolidating multiple assays...").*

We thank Reviewer 3 for noting this remainder of a prior manuscript draft. Mention of costs were removed at lines 241 and 604 as recommended.

Sincerely,

Melissa L. Smith, Ph.D.

Associate Professor

Department of Biochemistry and Molecular Genetics

Department of Microbiology and Immunology
