# Supplementary material for: Development and validation of HIV SMRTcap for the characterization of HIV-1 reservoirs across tissues and subtypes
Source: PLoS Pathog. 2026 Jan 13;22(1):e1013171. doi: 10.1371/journal.ppat.1013171 (PMC12851485; doi:10.1371/journal.ppat.1013171)
Supplement: Attachment — Submitted filename: HIV_SMRTcap_Methods_ReviewersCommentsOnly_R2_FINAL.docx [file ppat.1013171.s008.docx]

November 24, 2025

Dear Editorial Staff and Reviewers,

As corresponding author, I want to thank you and our reviewers for your kind words on our revised manuscript draft and for catching several issues left behind to get our manuscript, Sadri, et.al., *Development and validation of HIV SMRTcap for the characterization of HIV-1 reservoirs across tissues and subtypes*, publication ready. We have addressed all comments below in an itemized fashion, and we are hopeful that after incorporating these changes, the team will find it acceptable for publication in PLoS Pathogens.

*Itemized responses to editorial and reviewer comments are below. For ease of interpretation, editorial and reviewer comments are italicized and our responses are in* standard text:

**Reviewers' Comments:**

**Minor Issues: Editorial and Data Presentation Modifications**

Reviewer #2:

1. *Figure 7 is now an independent figure, while it was previously part of Figure 6, but there is no figure legend for the new Figure 7. Please align the figure legends and figures (move current Figure 6E-F legend to independent Figure 7 legend, and make current Figure 7 legend the Figure 8 legend).*

We thank Reviewer #2 for catching this! The figure legend for Figure 6 has been split, and the new figure legend for what is now Figure 7 can be found at Line 1106. The prior Figure 7 has been relabeled Figure 8, with the legend adjusted at Line 1112.

2. *There appears to be a missing arrow for the BACH2 integration clone for Figure 7B, Y9. The arrow is also missing in Figure 2B for chromosome 13 integration.*

Arrows have been added to both Figure 7B, Y12 (for ease of placement, compared to Y9) and Figure 2B, as highlighted in this comment.

3. *The significance asterisks are missing in Figure 3C.*

Significance asterisks have been re-added to Figure 3C.

4. *Newly included QVOA data in tables for Figures 6 and 7 have not been cited for the source of the data.*

We thank the reviewer for this point and have highlighted the original data publication (Ferreira et al.) in both legends for Figures 6 and 7 (Lines 1103 and 1110, respectively) to ensure that readers understand that these data were sourced from prior work.

5. *It seems a library of p88 of 8e5 cells was used for SMRTcap sequencing and comparison to LM-PCR in Figure 2C. If so, please include the sequencing read data in Table 3 and in a GenBank BioProject.*

We thank the reviewer for this comment. In fact, they have caught a small typo that clearly led to reader confusion. For the 8e5 passaged cells, we sequenced passages 2, 44, 86, and 112. We did not sequence passage 88. However, passage 86 data was mis-noted as “passage 88” twice in the text, and these typos have been corrected in the current “R2” version of the manuscript. Thus, no additional data needs to be represented or uploaded. We sincerely apologize for any confusion these typos may have caused.

6. *Figure 6A contains no Y chromosome in the Circos plot although these data are from the male patient P4.*

We thank Reviewer 2 for noticing this oversight; the Y axis has been added back to the figure.

7. *It appears that in response to reviewer comments, what were originally two proviruses integrated in chromosome 5 in Figure 6A were re-analyzed and designated as a single provirus/clone. This makes 6 proviruses, which are shown in Figure 6A and 6B, but text in line 345 of the manuscript clean copy states there are 7. Also, the gene given in line 350 (ENSG00000264058) does not match the gene in Figure 6B.*

We thank the reviewer for identifying these discrepancies between our original and revised manuscript drafts. The highlighted typos have been edited and the text amended (Line 348). Further, ENSG00000264958 in the text was converted to ENSG00000264058 as it should be (Line 354).

*8. Line 196 should direct to Table 3, and line 540 should direct to Table 2.*

Both suggested edits were incorporated on Lines 196 and 545, respectively.

*9. Table 1 still appears to contain the typo for the ID of SE365, which should be AB485649.*

We thank the reviewer for catching this and have removed the duplicated “5” causing this error.

*10. The methods for PDX mice should state 13 weeks post-engraftment or eight weeks post-infection in line 572, and inclusion of dose and route of HIV infection information in this section of the methods would be appropriate.*

We have altered the methods language here to make the timing of infection and tissue collection clearer, as per the reviewer’s recommendations (Line 579). Moreover, we have included added details regarding infection (Lines 576-577).

*11. Typos in line 538 (should be Alemtuzumab), 366 (should be ENSG00000288724 per figure 6D), and line 245 (between 0% and 75%); additional typos in lines 755 and 1075.*

All typos were addressed.

Sincerely,

Melissa L. Smith, Ph.D.

Associate Professor

Department of Biochemistry and Molecular Genetics

Department of Microbiology and Immunology
